# Botulinum Neurotoxin Diversity from a Gene-Centered View

**DOI:** 10.3390/toxins10080310

**Published:** 2018-08-01

**Authors:** Roger M. Benoit

**Affiliations:** Laboratory of Biomolecular Research, Division of Biology and Chemistry, Paul Scherrer Institute, Villigen CH-5232, Switzerland; roger.benoit@psi.ch; Tel.: +41-56-310-4703

**Keywords:** botulinum neurotoxin evolution, toxin diversity, selfish genes, gene-centered view, the role of botulinum neurotoxins in nature, carcass–maggot cycle, toxin architecture

## Abstract

Botulinum neurotoxins (BoNTs) rank amongst the most potent toxins known. The factors responsible for the emergence of the many known and yet unknown BoNT variants remain elusive. It also remains unclear why anaerobic bacteria that are widely distributed in our environment and normally do not pose a threat to humans, produce such deadly toxins. Even the possibility of accidental toxicity to humans has not been excluded. Here, I review the notion that BoNTs may have specifically evolved to target vertebrates. Considering the extremely complex molecular architecture of the toxins, which enables them to reach the bloodstream, to recognize and enter neurons, and to block neurotransmitter release, it seems highly unlikely that BoNT toxicity to vertebrates is a coincidence. The carcass–maggot cycle provides a plausible explanation for a natural role of the toxins: to enable mass reproduction of bacteria, spores, and toxins, using toxin-unaffected invertebrates, such as fly maggots, as the vectors. There is no clear correlation between toxigenicity and a selective advantage of clostridia in their natural habitat. Possibly, non-toxigenic strains profit from carcasses resulting from the action of toxigenic strains. Alternatively, a gene-centered view of toxin evolution would also explain this observation. Toxin-coding mobile genetic elements may have evolved as selfish genes, promoting their own propagation, similar to commensal viruses, using clostridia and other bacteria as the host. Research addressing the role of BoNTs in nature and the origin of toxin variability goes hand in hand with the identification of new toxin variants and the design of improved toxin variants for medical applications. These research directions may also reveal yet unknown natural antidotes against these extremely potent neurotoxins.

## 1. BoNT Variability

Botulinum neurotoxins (BoNTs), produced and secreted by *Clostridium botulinum* and some other clostridia [1,2,3], rank amongst the most potent toxins known [4]. The spores of clostridia are commonly present in the environment, for example in soil, water, and on foods [5]. In a highly diluted form, some BoNTs are widely used in medicine and cosmetics, for example under the trade name BOTOX [6,7]. Surprisingly, more than a century after the initial discovery of anaerobic soil bacteria that produce BoNTs [8], it is still not fully clear why these bacteria produce toxins that are so highly poisonous to humans, and why so many diverse BoNTs exist.

*Clostridium botulinum* bacteria belong to four different groups (I–IV), which can be considered as different species that have in common that they produce botulinum neurotoxins [9]. *Clostridium baratii* (Group V) and *Clostridium butyricum* (Group VI) are two additional species that also produce BoNTs [1,2]. 

A wide variety of serotypes and subtypes of BoNTs have been identified in nature [10,11,12], indicating that the changes in the toxins may indeed have evolved for adaption to specific environments. Changes in the amino acid sequence of BoNTs could result in higher toxicity towards specific vertebrate species. Mutations that increase the toxicity towards species living in the habitat of BoNT-producing bacteria would result in an increase in available nutrients, and hence in increased propagation of the bacterial strain and of the toxin gene cluster. 

At least seven serotypes (A–G) of botulinum neurotoxins (BoNTs) exist [13]. A serotype is defined as a toxin that can be neutralized with a type-specific antitoxin [14]. BoNT serotypes can further be subdivided into subtypes. A BoNT subtype is defined as having more than 2.6% difference in the amino acid sequence compared to known sequences [15]. Subtypes have been described for BoNT serotypes A, B, E and F [16]. 

**Figure 1:**
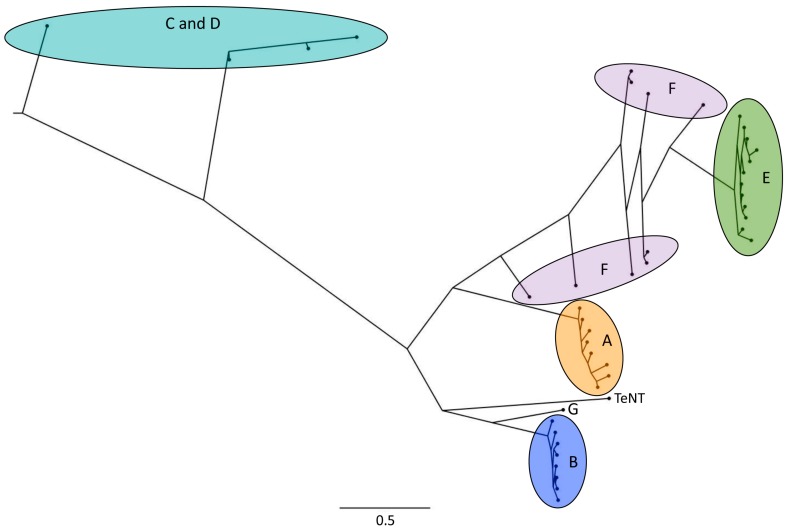
Phylogenetic tree of botulinum neurotoxin (BoNT) serotypes and subtypes, depicting the large diversity in the toxin amino acid sequences. The highlighted areas/letters indicate BoNT serotypes, the subtypes are shown as black dots. The scale bar indicates branch length (amino acid substitutions per site). TeNT = Tetanus neurotoxin. The tree was computed using PhyloBot [22] and the Figure was created using FigTree (http://tree.bio.ed.ac.uk/software/figtree/).

Serotypes A, B, E and F cause botulism in humans [4,10,17], but they are also toxic to animals. For example, BoNT subtypes A, E, F, and at higher doses also BoNT/C, were shown to be toxic to Zebrafish [18]. BoNT/A subtypes A1 and A2 are toxic to mice and rats [19] and BoNT/B1, B2, and B6 were shown to be toxic to mice [20]. Serotype C mostly causes botulism in birds, while BoNT/D can cause botulism in various animals [10]. According to a case report, mosaic toxin CD can cause botulism in laying hens [21]. No subtypes have been reported for serotype G. For BoNT/C and BoNT/D, mosaic toxins exist. For example, the mosaic BoNT/DC consists of a catalytic domain and translocation domain of BoNT/D and of a receptor-binding domain of BoNT/C [16]. A phylogenetic tree of BoNT serotypes and subtypes is shown in Figure 1.

The distribution of bacterial strains producing various BoNT serotypes differs substantially (reviewed in Ref. [23]): *Clostridium botulinum* that produce BoNT serotypes A, B, E, F and G can be encountered in soil as well as in marine and lake water sediments. Toxinotype A and B strains are widespread in the USA and are most abundant in neutral to alkaline soil. Toxinotype E strains are more often associated with water sediments or fish and wet soils. A special property of this toxinotype is its propagation in areas of low temperatures. It is prevalent in northern areas. Strains producing BoNT serotypes C and D are typically associated with cadavers of birds and other animals in many areas worldwide. Toxinotypes F and G have been detected in soil and water sediments, however their occurrence is rare compared to other types. 

Analysis of next-generation sequencing data is expected to provide more detailed insights into the distribution of the different toxin subtypes and serotypes and is also expected to reveal the existence of yet unknown toxin variants [10]. Next-generation sequencing allows sequencing of DNA in a much higher throughput than previously available methods [24]. Therefore, many more samples from soil or other environments can be tested for the presence of BoNT-coding genes, and their precise sequence can be determined. Recent genomics efforts have confirmed the correlation of BoNTs with sediments, soil, and animals [25]. In an analysis of several thousand publically available metagenome datasets covering multiple environments, the largest number of DNA sequences with similarity to the catalytic domain of BoNTs were detected in samples from these bacterial habitats, and the study furthermore unveiled gene fragments with similarity to botulinum toxins in the metagenomes of insect guts [25]. 

BoNTs form progenitor toxin complexes (PTCs) with nontoxic neurotoxin-associated proteins [26,27,28,29,30,31,32,33,34,35]. Non-toxic non-hemagglutinin (NTNH) protects the toxin from the acid environment in the stomach [26]. Progenitor toxin complexes from BoNT serotypes B–D and G and in some BoNT/A strains furthermore contain hemagglutinin (HA) proteins, which facilitate passage through the intestinal epithelial barrier [26,31,33,35,36]. 

**Figure 2:**
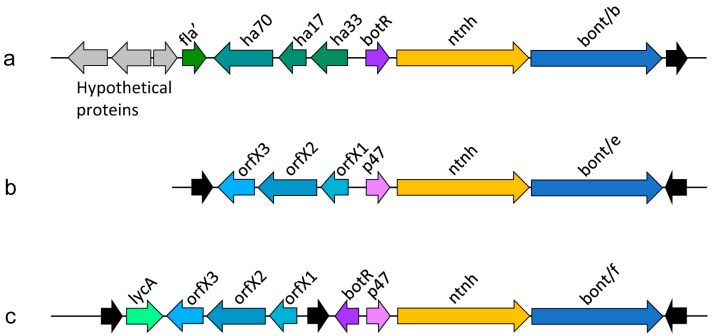
Examples of *Clostridium botulinum* neurotoxin gene clusters (schematic representations). (**a**) BoNT/B cluster located on a plasmid (pBf B); (**b**) BoNT/E cluster located on a chromosome; (**c**) BoNT/F cluster located on a plasmid (pBf F), an example of a cluster that comprises both, botR and p47. Summarized from Figure 3 of Ref. [9]. Flagellin (fla, partial gene), hemagglutinin (ha), regulatory protein (BotR), non-toxic non-hemagglutinin (ntnh), botulinum neurotoxin (bont), accessory proteins of unknown function (orfX, p47), lycA. The genes coding for BoNTs are clustered together with their accessory proteins and the clusters are often flanked by genes that facilitate horizontal gene transfer (complete or partial insertion sequence elements, shown as black arrows).

Botulinum neurotoxin genes typically cluster with genes coding for BoNT-associated proteins (Figure 2). The gene clusters usually comprise an operon containing an ntnh gene and the bont gene and an additional operon containing haemagglutinin proteins or, alternatively, orfX genes, whose function remains to be determined [37]. Recent findings indicate that OrfX1 and OrfX2 bind phosphatidylinositol lipids [38]. OrfX2 and P47 have structural similarity to proteins from the tubular lipid-binding (TULIP) domain superfamily, which are often involved in lipid binding [38,39]. The proteins may therefore have similar functions as HA proteins in the transiting of the toxin through the intestinal epithelial barrier [39]. 

A wide variety of gene combinations exist in bont gene clusters. For example, as a result of recombination in the ntnh gene, the bont/A1 gene can be associated either with ha+ or orfX+ toxin clusters, an arrangement that has not been observed for any other subtype [40]. More typically, specific bont genes can be found within distinct gene clusters [40]. 

A serotype B/A chimera of ntnh has also been described and is believed to have formed by recombination between a ntnh gene of a serotype B strain and a serotype A ntnh gene [9,40,41]. It has furthermore been suggested that the bont/A2 subtype has its origin in a recombination event between subtypes A1 and A3 [12,40]. Comparably, recombination between bont/F1 and bont/F2 resulted in bont/F6 [40,42]. Bont F6 is associated with Group II *C. botulinum* strains, while bont/F1 and F2 are associated with group I *C. botulinum* strains, suggesting that horizontal gene transfer was involved as well [40]. The reported recombination events between bont and bont-associated genes are too numerous to be described comprehensively here. The above examples show how horizontal gene transfer and recombination has contributed to BoNT variation.

The BoNT gene clusters are commonly embedded in mobile genetic elements or on plasmids or phages [40]. The genes coding for pathogenic factors evolve faster than other parts of the genome, which could indicate that adaptation of the toxin-coding DNA to new environments is important for bacterial survival [43]. Nevertheless, it remains unclear whether toxin production and variability truly results in an evolutionary advantage [10]. Popoff suggested that the production of BoNTs by clostridia does not result in a selective advantage over clostridia that do not produce toxins [23]. Even the possibility of accidental toxicity to vertebrates has not been excluded, as BoNTs could potentially have other, yet unknown functions, such as for example signaling or communication [10].

## 2. Botulism

The disease caused by BoNT poisoning is known as botulism. Botulinum neurotoxins are lethal at considerably lower doses than for example cyanide [44,45]. Through an enzymatic mechanism, a single molecule per cell may be sufficient for toxin action [44,46]. 

Botulism is a potentially fatal, neuroparalytic disease that causes flaccid paralysis of motor and autonomic nerves, which can result in respiratory failure [8,16,17,47]. 

The effects of BoNTs were initially observed in food poisoning outbreaks [8]. Precise descriptions of botulism date back to the early 1800s, but it wasn’t until 1895 that *Clostridium botulinum*, the pathogen responsible for the poisonings, was discovered [8]. While early observations of botulism involved contaminated meats or fish, an outbreak in 1904, caused by canned beans, led to the discovery of a different strain of *Clostridium botulinum*, which produced a different BoNT serotype [8]. Today, at least seven serotypes, BoNT A–G, are known, of which four (A, B, E and F) cause botulism in humans [17]. Food-borne botulism, resulting from ingestion of improperly canned or pickled foods, is caused by pre-formed toxins contained in the foods [5,16,48]. Foods are often contaminated with *C. botulinum* spores. Canned, protein-rich foods, such as meats or vegetables, provide an ideal anaerobic environment for spore germination and bacterial growth, which results in toxin production by the bacteria and hence contamination of the foods with pre-formed toxin [49]. Interestingly, food-borne botulism outbreaks in humans typically arise from processed foods that are not found in nature. Therefore, these contaminated foods are unlikely to have played a role in the evolution of these toxins.

The bacteria can also populate anaerobic regions of the intestine. Infant botulism is caused by the ingestion of foods, for example honey, that are contaminated not with toxin, but with *Clostridium botulinum* spores, resulting in intestinal colonization and toxin production [16,48,50]. Wound botulism is yet another form of the disease, caused by wound contamination with *Clostridium botulinum* spores, followed by colonization of the wound with *C. botulinum* and toxin production [5,16,47,48]. 

## 3. The Highly Specific Mode of Action of BoNTs

**Figure 3:**
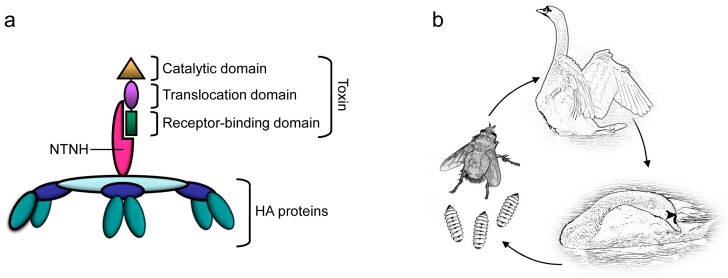
(**a**) Schematic representation of the toxin architecture: Botulinum neurotoxins (BoNTs) appear like precisely engineered nanomachines: The toxin itself comprises three domains that recognize the target neurons (receptor-binding domain), allow the toxin to enter cells (translocation domain), and cleave (catalytic domain) proteins in the cell that are required for neurotransmitter release, resulting in flaccid paralysis. In progenitor toxin complexes (PTCs), non-toxic neurotoxin-associated proteins, comprising non-toxic non-hemagglutinin (NTNH) and sometimes also hemagglutinin proteins (HA), protect the toxins from the harsh environment in the stomach and facilitate passage through the intestinal epithelial barrier into the bloodstream; (**b**) The carcass–maggot cycle provides a possible rationale for the production of botulinum neurotoxins in nature: Animals often carry *Clostridium botulinum* in the intestine, without developing any symptoms of botulism. If the animals die of any cause, the carcass provides a protein-rich, anaerobic environment that allows the bacteria to grow and produce large amounts of toxin. The toxin accumulates in fly maggots feeding on the carcass, because invertebrates are not vulnerable to the toxin. Animals feeding on the maggots die, starting a new cycle of large scale *Clostridium botulinum* production.

The sophisticated mechanisms used by BoNTs to reach neurons and to block neurotransmitter release (summarized in Figure 3a) make it seem highly unlikely that BoNT toxicity to humans or other vertebrates could be a coincidence: BoNTs and their associated proteins act like precisely engineered nanomachines equipped with mechanisms that allow them to survive the harsh conditions in the stomach, to enter the bloodstream through the intestinal epithelial barrier, and to recognize [51,52,53,54,55,56,57,58,59,60,61] and enter [62,63,64,65] peripheral nerve terminals, which are their target cells [17,26,62,66,67] (Figure 3a). BoNTs cannot cross the blood-brain barrier [16,68] but it has been shown that BoNT/A and E can under some circumstances reach the central nervous system by axonal retrograde transport [68]. Toxin action at peripheral nerve terminals is however sufficient for the extreme toxicity observed in nature.

Once BoNTs have entered a neuron by receptor-mediated endocytosis, a pH-induced structural change of the translocation domain of the toxin results in channel formation [69]. The protease domain is translocated through the channel and released into the cytoplasm, where it specifically cleaves one of the SNARE proteins that are required for vesicle fusion [17]. As a result, neurotransmitter release at the neuro-muscular junction is blocked, resulting in flaccid paralysis. The numerous specialized steps that are required for toxin action indicate that natural selection played a role in the development of BoNTs.

## 4. BoNTs as a Natural Tool for Bacterial Mass Propagation

A plausible explanation for the energy-consuming toxin production by the bacteria becomes apparent when looking at botulism in the wilderness, where processed foods are not involved. BoNT-producing bacteria appear to act as parasitic predators, by killing a host and then feeding on it [70]. Birds and fish (and mammals) often contain *Clostridium botulinum* in the intestine but do not show any symptoms of botulism [43]. When an animal dies from another cause than the bacteria, the spores germinate in the anaerobic carcass and the bacteria propagate and produce BoNTs. Anaerobic carcasses in nature provide similar ideal growth conditions for *Clostridium botulinum* as do improperly canned, protein-rich foods in the civilized world. 

A special way of toxin-induced bacterial propagation has been described for waterbirds and fish, whose diet includes maggots. BoNTs are not toxic for invertebrate animals [43]. In avian botulism, necrophagous flies play an important role. They lay eggs on carcasses. If a carcass contains botulinum neurotoxins, the BoNTs accumulate in the fly maggots feeding on the carcass, without affecting the maggots themselves [43]. In this way, BoNTs enter the food web. Birds feeding on the maggots die from the toxin, resulting in an amplification of bacteria, toxin, and spore production [71]. This amplification cycle is known as the carcass–maggot cycle [43,71] (Figure 3b). 

## 5. The Gene-Centered View of Evolution—Selfish Genes

In his book “on the origin of species by means of natural selection” [72], Charles Darwin postulated that species evolve through natural selection, a theory that is now widely accepted. The species or organisms were typically regarded as the evolving entities. The gene-centered view of evolution arose much later, as more became known about the molecular mechanisms of genetic inheritance, and received much attention through the book “the selfish gene” by Richard Dawkins [73]. In a gene-centered view of evolution, the DNA itself, rather than the organism containing the DNA, is considered to be the evolving entity [74,75,76,77]. 

Using the molecular machinery of cells, DNA molecules are able to replicate and they determine the phenotypes of their “host” cells through the proteins that are coded by their genes or gene clusters. In a gene-centered view, evolution favors genes or gene clusters that persist over an extensive period of time over many generations [77,78]. If functionally related genes are clustered in operons, the proximity of the genes enhances the chance that a functional entity containing all the components coding for a selectable phenotype are transferred to a new host together [78,79]. In this way, functional gene clusters can persist in a new host even if their original host becomes extinct [78,80]. To be stable and successful in evolution and to accumulate, a replicating entity either has to be long-lived, has to replicate more frequently than others, or has to be conserved through high-fidelity replication [77].

In humans, the concept of selfish or “immortal” genes can be explained as follows [77]: As a result of genetic recombination, each individual is unique and in the context of evolution short-lived. It is the genes or gene clusters that successfully replicate which are long-lived over many generations. In a gene-centered view, such gene clusters form the entities that are relevant for evolution. 

Due to the tight link between genotype and phenotype, for many genes coding for normal cellular functions, it is difficult to differentiate whether or not they propagate in a selfish way. In some organisms however, specific gene clusters have been identified that behave in a way that strongly suggests that they indeed act as selfish entities. Gene clusters coding for toxins and their corresponding antidotes are examples of selfish genes. Recently, it was shown that a selfish genetic element in a strain of the nematode *Caenorhabditis elegans* codes for a maternally deposited toxin that causes embryonic lethality and for the zygotically expressed antidote [81,82]. Embryonic development is normal in strains lacking the toxin–antitoxin genetic element and in strains containing both, the toxin and the antitoxin gene. When the two strains are crossed, in the F2 generation, only the offspring that inherited the gene coding for the antidote against the maternally deposited toxin survives. Because the genetic element is not needed by the organism, but the organism becomes “addicted” to the gene cluster once it is present, it seems likely that the genetic element acts as a selfish gene. Similarly, recent evidence in *Schizosaccharomyces kambucha* and *S. pombe* suggests that meiotic drivers are selfish genetic elements that encode a toxin that kills gametes and an antidote that rescues them [83]. Furthermore, a wide range of toxin–antitoxin systems have been described in bacteria, typically comprising a stable toxin and a labile antitoxin [84]. Often, the toxin–antitoxin systems are located on mobile genetic elements and may act as selfish gene clusters. If a bacterium for example loses a plasmid coding for the antitoxin (and toxin), the cell dies, because the antitoxin is degraded faster than the toxin.

## 6. BoNT-Coding DNA from a Gene-Centered View

Toxin–antitoxin systems are good examples of genes that have been extensively studied in the context of selfish behavior. A major difference to BoNT-coding gene clusters is that the components of the toxin–antitoxin systems act within the same organism, while botulinum toxins and toxin-associated proteins are secreted and act on different organisms. The link between the toxin-coding gene clusters and the toxins is indirect, through the proximity of the toxin-producing cells and the secreted toxins, which can provide additional nutrients. When a toxin gene cluster moves to a new host, diversification may lead to the formation of a toxin variant that is optimally active in species living in the environment of the new bacterial host. 

Several observations related to BoNT-coding gene clusters can better be explained by a gene-centered view than by a classical view of evolution. For example, the production of BoNTs is not as strongly connected to a specific species as was originally expected. *Clostridium botulinum* was discovered after an outbreak of food-borne botulism resulting from the ingestion of contaminated pickled and smoked ham in 1895 [8]. Botulinum neurotoxin production was historically viewed as a property of a specific bacterial species. Originally, the species *C. botulinum* included all known BoNT-producing bacteria, later, *C. botulinum* was divided into four different groups designated I–IV, which represent separate species [9]. *C. baratii* and *C. butyricum* strains also produce BoNTs [9]. Moreover, a BoNT-like toxin has been identified in the bacterium *Enterococcus faecium* [85] and a BoNT homolog has been reported in *Weissella oryzae* [86,87]. 

In bacteria, conjugation (DNA transfer from cell to cell), transduction (DNA transfer through bacteriophages), and transformation (uptake of DNA from the surroundings) as well as subsequent recombination and transposition can result in changes in the genotype [88]. Genes coding for BoNTs and related genes coding for important BoNT-associated proteins often co-localize on gene clusters that are flanked by mobility-enhancing sequences [9,14,89]. In a gene-centered view, proximity of functionally related genes is a prerequisite for the formation of a replicating entity that has a high probability of being stable over many generations, because genes that are close together have a higher chance of being transferred together in gene transfer events [77]. The observation that BoNTs or bont-like genes are present in several distinct species and even genera, and that horizontal gene transfer and DNA recombination are believed to have strongly contributed to toxin variability [10,37,40,85], leads to the impression that BoNTs, and also their evolution, are not inseparably linked to a specific bacterium. It rather appears that in toxin evolution, natural selection favors functional toxin gene clusters that use suitable bacteria as their hosts, and if replication is successful in a specific host and environment, both, the host and the toxin cluster propagate efficiently. 

Another aspect of botulinum neurotoxin behavior that is difficult to explain by a traditional view of evolution is that toxin production does not result in a direct competition with other bacterial species. If the species themselves were the evolving entities, toxin-producing clostridia would be expected to prevail and propagate at the cost of non-toxigenic clostridia. BoNTs do however not appear to target clostridia or other bacteria that may compete for food sources. Conversely, through their toxins, BoNT-producing clostridia can produce carcasses that increase the supply of nutrients also for other bacteria. As a result, there seems to be no direct, toxin-related competition between BoNT-producing clostridia and non-toxigenic clostridia or clostridia producing other BoNT variants. Furthermore, floating algae and decomposing plants can also provide an anaerobic environment required by *Clostridium botulinum* for growth [43]. Therefore, the bacteria are not dependent on vertebrate hosts. However, bacterial growth has been suggested to be much more efficient in decaying animals than in decaying plants [71]. The amplification cycle is boosted by the extreme potency of BoNTs. Botulism spreads in a similar way to an infectious disease through secondary poisoning [71]. The toxin produced by bacteria in infected bird carcasses can lead to poisoning of additional birds, using maggots as the vector [71]. BoNT-production can hence trigger an amplification cycle that results in large-scale production of bacteria and spores, as well as the bacterial DNA, including the toxin gene clusters. In environments that are inhabited by vertebrate species that are vulnerable to the toxins, toxin production does of course result in increased availability of nutrients to the bacteria, which can lead to transient, local bursts of proliferation and possibly distribution of the bacteria [10]. The proliferation of the bacteria parallels large scale production of toxin-coding genes. Assuming that these toxin clusters replicate as selfish genetic elements, the benefit to the bacterial multiplication could be viewed as a collateral effect. A beneficial effect to the host is not in conflict with self-propagation of a genetic element. However, non-toxigenic bacteria that populate the same habitat also profit from the additional nutrient sources. BoNT-induced carcasses and the carcass–maggot cycle increase the availability of nutrient-rich anaerobic environments [10] not only to toxigenic clostridia, but for anaerobic bacteria living in the same habitat in general. Toxins that target other bacteria in the struggle for nutrients would more specifically favor survival of a certain bacterial species over another. 

If natural selection of BoNTs would act on the organism, a specific toxin would be expected to be linked to specific clostridium species. Recent evidence however shows that some clostridia produce more than a single serotype of toxin [40] and there is clear evidence that toxin-coding gene clusters can shuttle between different bacterial species by horizontal gene transfer [9,14,89], and that horizontal gene transfer and recombination play an important role in toxin evolution [10,37,40,85]. It has previously been speculated that BoNTs may derive from a viral, rather than from a bacterial origin [90]. BoNT-coding DNAs may hence act as independent genetic elements that promote their own replication, akin to commensal viruses [91], using the machinery of the host for toxin production and DNA replication. Recent evidence from genomic and phylogenetic analysis supports the view that BoNTs have evolved separately from clostridia, and that the diversification of BoNT serotype lineages, which is currently poorly understood, could be explained by co-evolutionary diversification of host and pathogen [92]. Sequence differences between some BoNT serotypes are very large, while the variation within subtypes is much more subtle [92]. Amino acid sequence differences between known subtypes of a BoNT serotype range between 1.5% to 32.6% [93], although recent nomenclature suggestions state that newly identified sequences should be designated a new subtype if they differ by more than 2.6% to known sequences [15]. Subtype differences are rather large in serotypes A, C, D, and F and rather small in serotypes B and E [93]. 

The large number of toxin cluster replication events taking place in a bacterial host which propagates in a carcass may result in DNA replication errors that increase diversity in the BoNT sequences. Accumulation of mutations that do not affect toxin function may contribute to the emergence of new serotypes or subtypes. The same holds true for mutations that increase toxicity towards a specific vertebrate species that lives within the habitat of the bacterial host of the toxin cluster. In the latter case, the more potent toxin may even result in a stronger amplification cycle and in increased accumulation of the toxin type. Interestingly, the toxin serotypes C and D and mosaics thereof, which are typically associated with birds and are hence involved in the carcass–maggot cycle [94], branched off very early from a common ancestor of all toxin serotypes (Figure 1). The other serotypes hence diversified from a serotype C or D-like common ancestor that may have been involved in the carcass–maggot cycle.

Tetanus toxin, which is closely related to botulinum toxins, has not diversified within the toxin sequence [95]. It is difficult to extrapolate why the tetanus toxin sequence remains more constant. In the context of selfish genes, tetanus toxin genes can be viewed as a replicating unit that is successful because it is conserved over many generations through high-fidelity replication.

Research addressing toxin variability goes hand in hand with a profound understanding of the natural processes, such as natural selection, that drive the emergence of novel toxin variants.

The comprehension of the factors driving the evolution of BoNTs is complicated by the circumstance that BoNT-coding genes can be located on mobile plasmids or on the bacterial genome, and that co-evolution of clostridia and BoNT-coding genes may also have played a role. Evolution works at the level of the organism, but also at the molecular level [96]. The host and the mobile genetic elements have very different generation times. For similar reasons, it remains difficult to understand co-evolution of viruses with their hosts [91]. Many viruses live in a commensal symbiotic relationship with their host and can be considered as selfish elements [91]. 

The observations described above support the view that in BoNT evolution, the BoNT-coding gene cluster, rather than the organisms hosting it, is the final beneficiary of toxin activity, and that these gene clusters may self-propagate as selfish genes in a suitable host. Because the genotype and the phenotype of an organism are tightly linked, it is difficult to clearly differentiate between selection for an organism versus selection for a gene cluster. Through next-generation sequencing projects, the number of available genome sequences is constantly growing. Systematic analysis of such genomic data for the presence DNA fragments that have been carried over from previous toxin hosts to new toxin hosts in the process of horizontal gene transfer may in the future allow a more precise view on the identity and number of former hosts of a specific toxin cluster in the course of its evolution. In a similar way, analysis of non-toxigenic bacteria for leftovers of toxin-cluster DNA could provide valuable information on the frequency of horizontal gene transfer and its importance for BoNT evolution. 

## 7. Conclusions

Food-borne botulism likely affected human populations already in ancient times [8]. Before the botulism-causing bacterium *Clostridium botulinum* was discovered in 1895, cases of botulism may have been mistaken for other diseases or intoxications, therefore, there are no clear records of botulism from earlier years [8]. Interestingly, clostridia that do not produce BoNTs do not seem to have a selective disadvantage [23]. A possible explanation could be that a wide variety of clostridia live in the same habitat, and that non-toxigenic strains profit from the toxigenicity of other strains. Alternatively, this observation could be explained by a gene-centered view presented here. 

BoNT research has strongly focused on the use of the toxins for clinical or cosmetic applications. Many open questions about the natural rationale of these toxins, and about the factors responsible for their evolution, remain. Future studies addressing the role of BoNTs in the wilderness, along with next-generation sequencing, as recently suggested by Montecucco and Rasotto [10], are likely to result in the discovery of new toxin variants, possibly with improved clinical properties. Recently, DNA fragments with significant sequence similarity to BoNTs have been identified in the metagenome from termite gut through the analysis of metagenomic sequencing data [25], showing the high potential of next-generation sequencing approaches for BoNT research. This field of study may in the future reveal yet unknown natural antidotes against the extremely potent neurotoxins.
